# ‘Semen Contains Vitality and Heredity, Not Germs’: Seminal Discourse in the AIDS Era

**Published:** 2006-12

**Authors:** Sharful Islam Khan, Nancy Hudson-Rodd, Sherry Saggers, Mahbubul Islam Bhuiyan, Abbas Bhuiya, Syed Afzalul Karim, Oratai Rauyajin

**Affiliations:** ^1^ Social and Behavioural Sciences Unit, Public Health Sciences Division, ICDDR,B, GPO Box 128, Dhaka 1000, Bangladesh; ^2^ School of International, Cultural and Community Studies, Edith Cowan University, Perth, Western Australia; ^3^ Department of Dermatology, Holy Family Red Crescent Medical College Hospital, Dhaka, Bangladesh; ^4^ Faculty of Social Sciences and Humanities, Mahidol University, Bangkok, Thailand

**Keywords:** Semen loss, Sexuality, Sexual health, Sexually transmitted infections, Human immunodeficiency virus, AIDS, Bangladesh

## Abstract

Perspectives of public health generally ignore culture-bound sexual health concerns, such as semen loss, and primarily attempt to eradicate sexually transmitted infections (STIs), including human immunodeficiency virus (HIV). Like in many other countries, sexual health concerns of men in Bangladesh have also received less attention compared to STIs in the era of AIDS. This paper describes the meanings of non-STI sexual health concerns, particularly semen loss, in the masculinity framework. In a qualitative study on male sexuality, 50 men, aged 18–55 years, from diverse sociodemographic backgrounds and 10 healthcare practitioners were interviewed. Men considered semen the most powerful and vital body fluid representing their sexual performance and reproductive ability. Rather than recognizing the vulnerability to transmission of STIs, concerns about semen were grounded in the desire of men to preserve and nourish seminal vitality. Traditional practitioners supported semen loss as a major sexual health concern where male heritage configures male sexuality in a patriarchal society. Currently, operating HIV interventions in the framework of disease and death may not ensure participation of men in reproductive and sexual health programmes and is, therefore, less likely to improve the quality of sexual life of men and women.

## INTRODUCTION

Dominating perspectives of public health often ignore cultural meanings of sexual health concerns, such as semen loss, and mainly work to prevent sexually transmitted infections (STIs)/HIV in the framework of disease and death. Traditional health practitioners deal with psychosexual health concerns of men but their treatments are often considered inappropriate by dominant doctrines of biomedical science. Ultimately, men are trapped and puzzled about how to get access to appropriate information and treatment options.

In the changing paradigm of reproductive and sexual health, non-STI sexual health concerns of men cannot be ignored as their ‘personal’ or ‘psychological’ problems. The sociocultural meanings of non-STI sexual health problems of men have received little attention with some notable exceptions in South Asia and elsewhere (1–5) In South Asian countries, researchers have demonstrated that men are worried about semen loss and other non-STI sexual health problems (1, 2, 4–9). Studies revealed similar concerns of non-STI sexual health anxieties among European and American male populations (3, 10). ‘Western’ biomedical scientists have investigated psychosexual problems in clinical settings (11, 12) but have ignored the social aspects of these problems (13).

In the Indian state of Orissa, a major male concern reported was *dhatu padiba*, the passage of ‘white discharge’ (perceived as semen) through urination and defaecation due to ‘thinning of semen’ (1). About two-thirds of Muslim slum dwellers in Mumbai, India, reported wasting of semen in the form of ‘white discharge’ through nocturnal emission, urination, or defaecation (5). These men believed that reduced quantity and thinning of semen led to *kamjori* (sexual weakness), a serious health problem.

A population-based survey of prevalence of STIs conducted in Matlab, a rural area of Bangladesh, revealed a low prevalence of STIs among men but the prevalence of psychosexual problems was comparatively higher (1). The survey reported that 17% of men suffered from psychosexual problems, including premature ejaculation, impotence, ‘dissatisfaction’ with sexual intercourse, difficulties in maintaining an erection, and nocturnal emissions. During the first year of the establishment of male sexual health clinics at Matlab, 41.5% of adult men attended the clinic with psychosexual problems reflecting similarities to that of the Indian context (14).

As in other South Asian countries, in Bangladesh, neither the public nor the private modern health sector accords importance to non-STI sexual health concerns of men, and consequently they have not been incorporated into service priorities. This results in missed opportunities to access men practising risky behaviours and to render appropriate services to improve the quality of sexual and reproductive health of men (and women).

This paper offers deeper meanings of semen loss and related concerns of Bangladeshi men and analyzes findings in the broader context of a sociocultural and masculinity framework with an expectation that policy planners and programme managers will re-visit the current paradigm and re-design STI interventions to interact with culture-bound concerns of men about semen loss.

### The context: men, semen, and sociomedical history

The importance of semen is deeply rooted in the historical and sociocultural belief system of the Indian sub-continent. Results of studies of the Indian context suggest that men consider that semen loss often leads to both sexual and non-sexual health problems (2, 4–7, 15–22). The complaint of ‘whitish discharge’ as semen has questionable scientific corroboration (23). Researchers have described it as a culture-bound syndrome, specifically named as *dhat syndrome* in the Indian sub-continent (6, 7, 15, 16, 20–22). The *dhat syndrome* has been incorporated in Annex 2 (Culture-specific disorders) of the ICD-10 Diagnostic Criteria for Research (24). Findings of this study revealed that concerns of Bangladeshi men about semen loss are also culture-bound syndromes, and we agree that these conditions: (a) are not seen in the West; (b) are not mere variants of well-recognized psychiatric disorders; (c) have geographically-defined prevalence; and (d) are determined largely, at least in the symptomatology, by the beliefs and assumptions prevalent in the native culture (25).

By observing cross-cultural similarities regarding semen loss, Herdt wrote of Sambian men, “the psychosocial phenomenon of semen depletion, a culturally-transmitted belief that men's sexual contacts rob and empty them of their semen, maleness, and eventually life itself is known from pre-modern and preliterate societies, including our own” (26). The concept of semen loss across culture has different meanings. Herdt reported, “semen is the substance closet to breast milk, and it provides the next sort of [‘biological’] push that boys require. Elders reiterate that boys should ingest semen every night, as if it were breast milk or food (27).”

A universality of linguistic dynamics regarding semen is seen in South Asia. In Hindi, semen is called *virya* which means vigour. Men's eternal force of life and survival is believed to be conserved in semen. The ‘excessive’, ‘unnatural’, or ‘immoral’ semen loss can negatively impact on health in general and sexual health in particular (28–30). The word *dhat* has been found in Sanskrit, from a word *dhatu* which indicates semen. The notion of a human body as a biological device, in which diseases originate, has limitations in terms of ignoring the truth that the human body also has social and cultural realities where perceptions of health and sickness are grounded. Many illnesses without any organic pathology are culturally produced and bound among men and women across societies (3, 31–36). The ethnomedical perspectives of *ayurvedic* medicine (37) have potential to explain health and illness, including loss of genital secretions through a ‘cultural prism’ through which men and women traditionally view the self and the body throughout South Asia (36).

## MATERIALS AND METHODS

Men from both urban and rural settings of Bangladesh are not conventionally identified as members of any known sub-populations involved in risk behaviours. We have discussed the sexual lives of these men, particularly semen-related issues in a phenomenological qualitative study. We collected mens’ ideas about sexual health, particularly semen loss as it relates to sexual health and the well-being of men. We explored concerns of men about semen, its storage and loss, the quality and quantity during ejaculation, the way semen is lost, and other related worries. We interacted and spent a considerable amount of time with our informants and noted their worries and cultural understanding about semen through in-depth interviews and informal discussions. Thus, this paper draws on both ethnography and phenomenology to gather ‘deep’ information about a phenomenon as perceived by an ‘actor’ in a particular societal setting (38–41).

Two research assistants and the first author of this paper collected and managed qualitative textual data. Fifty men, aged 18–55 years, from diverse sociocultural, economic, educational and occupational backgrounds from an urban and a rural area of Bangladesh were selected based on their willingness to be interviewed. Five modern (allopathic doctors, of them two were STI specialists) and five traditional medical practitioners (e.g. *ayurvedic*, *unani*, *mogha*, homeopathic, and *kabiraj*) were interviewed as key-informants. Half of the interviewed men were married. Thirty percent had completed 10–16 years of education, and about 48% had no institutional education.

An open-ended interview guideline was used with flexibility for incorporating issues raised relating to the research topic for further exploration. Each interview took, on average, two hours. On a few occasions, we had to arrange several sessions to complete one in-depth interview. We analyzed metaphors described by participants reflecting their experiences and tensions relating to semen. These metaphors, useful in sexuality research, indicate the “point of connection between personal experience and cultural context” (42). Referred cultural scripts of participants, such as advertisements for traditional practitioners on sexual health issues, were also analyzed.

The participants often felt uncomfortable in providing written consent while discussing personal and intimate issues of their sexual lives. This formality appeared as a barrier to developing mutual trust and rapport with the participants. Therefore, verbal affirmation to be interviewed from an adult participant was considered appropriate to begin an interview. This verbal testimony was tape-recorded each time at the beginning of an interview.

Initially, we recruited professional translators to translate the tape-recorded interviews. However, we observed that the translated English version of the interview-texts lost the essence of sociolinguistic meanings and the context of psychosocial concerns of men regarding semen. We decided to transcribe the recorded interviews in the exact form by retaining special local terms and metaphors. Interpretation of data was manually performed by inter-subjective interpretations through examination of various interview-texts and field-diaries. We not only discussed the complex issues with the members of the research team, some informants and key-informants also participated in the analysis process. We made careful, repeated, and systematic reviews of the transcripts linking them to the research questions and other relevant emerging views as suggested by several scholars (41, 43–45). Note-cards were used for identifying prominent themes, logical connections, clarifications, or relevant comments to assist in explaining similar statements made by informants. We identified and categorized emerging thoughts regarding semen. This process included the identification of salient themes and sub-themes, recurring ideas, meanings or languages, and logical relations linking people and their milieus. Atypical or diverse data were not disregarded, but presented for analysis.

## RESULTS

Findings on various diverse perceptions of men about semen were grouped under the following themes: (a) disease and well-being, (b) masculinity, and (c) perspectives of medical practitioners. Perceptions of men were inter-linked with each other within a broader construction of masculinity.

### Seminal discourse: loss, disease, and well-being

No participant was concerned about the contamination of semen with HIV or other STI ‘germs’. The majority of men did not believe that semen contained viruses which could transmit infections to women. Men believed that the vagina of female sex workers was the ‘main’ source of infections. Some men claimed that men were not always responsible, rather, in many instances, women were also unfaithful. The common statement was, “men are smart and knowledgeable, they know how to protect them, but women are often inexperienced and depend on men for their protection.” These ideas reflect a gender-subordinated male supremacy of a patriarchal society of Bangladesh where men are considered ‘sexually expert.’

I do not think that men can transmit infections to women. Rather, men get infections from ‘bad’ women. There is no chance to get infection from faithful wife. However, if wives are unfaithful, they may transmit germs to husbands. Semen contains heredity, not germs.

In discussing sexual concerns, some men stated that ‘dilute’ semen was an indication of ‘sexual weaknesses,’ a common symptom and consequence of sexual diseases. Some young men sought treatment from traditional practitioners for their perceived *jouno rog* (sexual disease) relating to dilute semen. Their conditions were diagnosed as *dhatu durbolota* (literally means ‘weakness of semen’ which ultimately results in ‘sexual weakness’), a disease resulting from semen loss due to ‘excessive’ masturbation. Along with traditional medicines, men were advised to take protein-rich diet (e.g. eggs, meat, and milk), onion, and pumpkin to increase the concentration of semen.

Semen discharged through sexual intercourse, particularly within marital context, was described as normal. Ejaculation through sexual intercourse, particularly in a ‘legal relationship’ (i.e. with a wife), was described as ‘natural’, ‘healthy’, and ‘real’. The construction of ‘real’ sex in the domain of hetero-normative sexual cultures of Bangladesh encourages men to internalize the belief that semen should be ejaculated only within heterosexual intercourse. All other forms of ejaculations are considered ‘illegal,’ ‘unproductive’, and a ‘loss.’ Any loss of vital energy of the male body not only brings negative setbacks for men's health, but is also harmful to society. Thus, semen loss has societal meanings beyond physical bodies towards masculine discourse of male lives.

In a sexually silent society, a physiological phenomenon, such as nocturnal emission, is culturally labelled as shameful. Men described *swapno-dosh* (nocturnal emission) and *hasto-moithun* (masturbation) as two major ways of semen loss in their lives. Although all men experienced an involuntary emission of semen while sleeping (i.e. nocturnal emissions), most thought that this resulted from sexual diseases and sexual ‘weakness.’ The Bangla word *swapno* means dream and *dosh* means a fault. Therefore, the event of *swapno-dosh* is culturally labelled as a fault, connoting negative impressions as it indicated ‘unmet’ sexual desires of ‘sexually crazy’ men, who were imagining sexual relations with females while sleeping. The first experience of nocturnal emission indicated that someone had reached adulthood and was capable of ejaculating semen during a dream. This involuntary event placed men in a shameful situation as one's sexual maturation and stimulation could not be made public by ejaculating on the bed sheet. This denoted that, even after reaching adulthood, ‘good’ men are not to imagine sex. Many traditional practitioners advised men that nocturnal emissions could weaken their seminal strength.

Masturbation was the most secret sexual pleasure in the life of men and was reported as the first experience of voluntary ejaculation. All men spoke of masturbating, although most felt shy in expressing the action. Acknowledging social and religious disapproval, most men reported masturbation as an unavoidable reality in their lives. Most described it as a ‘bad habit’. The notion of ‘excessive’ masturbation varied among and between men. Unmarried men expressed concerns about masturbation in terms of ‘excessive’ semen loss, reduction in sexual potency, and deformed sizes and shapes of their penis. Findings revealed the following two major propositions:

- Masturbation=not a ‘real’ sexual activity=against the ‘nature’=perversion=religious sin=non-reproductive=useless expenditure of bodily energy through semen=harmful to general health=physical weakness=memory loss=loss of physical beauty.
- Masturbation=semen loss=depletion of semen reservoir=thinning of semen=lack of sperms=infertility.

Men believed that their general ill-health was related to semen loss, particularly through ‘unnatural’ ways (e.g. masturbation and nocturnal emissions). These men thought that semen loss caused their physical weakness, fatigue, palpitations, loss of interest, headache, abdominal pain, forgetfulness, darkness around eyes, and giddiness.

Some participants were confused about pre-ejaculatory fluid during sexual excitement. Young men stated that they had a secretion resembling *vater mar* (white discarded sticky watery substance at the end of rice-cooking) or *chuner pani* (whitish water of lime taken with betel leaf). They observed that this ‘whitish discharge’ before penetration occurs, sometimes without any sexual stimulation. Some reported discharging ‘whitish secretion’ when they felt sexual excitement, resulting in loss of their sexual erections and interest.

When I am sexually excited, some ‘whitish substance’ comes out through my penis. It looks like semen. However, I am not sure what it is. I am afraid and often lose my erection. Why this fluid comes before penetration? I am unmarried, so what will be my future? Now whenever I think about sex, it comes out. I am scared.

This man probably experienced the secretion of pre-ejaculatory fluid, a normal physiological response to sexual stimulation. However, due to lack of information and understanding of sexual physiology, men often reported ambiguous complaints. Men claimed that sexually-‘weak’ men could not control their ejaculation. Some stated that ‘whitish secretion’ was not semen, rather something else that was discharged due to some sexual disease and sexual weakness.

### Seminal discourse: masculine vitality

Men from all backgrounds considered semen the source of physical, sexual and manly strength. Semen of a healthy and ‘sexually strong’ man should be thick and milky-white. Any deviation, such as perceived ‘liquidity’ or ‘discoloration’ of semen, was threatening to masculine sexual potency in terms of sexual ‘performance’ of men and fertility. By the term sexual ‘performance’, men who paricipated in this study indicated being involved in prolonged sexual intercourse from penetration until ejaculation with a reported range between 20 and 30 minutes. Such prolonged intercourse is perceived necessary for providing sexual pleasure to women, and this has to be accredited by women. Thus, semen loss was seen as damaging to sexual performance of men as a man with ‘bad’ sexual performance is less masculine. Some men considered their semen ‘diluted’ and of ‘watery colour,’ indicating ‘bad’ quality, particularly for becoming fathers. One unmarried young man stated: “I think my body has deficiency of important nutrients, for which the quality of my semen is ‘bad’ which may lack an adequate number of *sukkro* (sperm). This can seize my power of impregnating my wife. You know that infertile men are not real men.”

Most men described sex as the way of spending physical energy through ejaculation of semen. Men claimed that 40 drops (the range was between 10 and 100 drops) of blood are required to form a single drop of semen. A common statement echoed: “that is why after ejaculation, I feel very tired, and I sleep within few minutes as I spend my physical energy through discharge of semen.”

Men believed that young men could produce semen rapidly because of their age and greater intake of food. Older men suffered from ailments (for example, diabetes and hypertension) that prevented them from taking nutritious (eggs, meat, milk, ghee, and butter) and sufficient amounts of food. Therefore, with aging, the production of semen is diminished and, as such, ‘manly strength’ declines making a man non-masculine.

Some men believed that poor people, who had less access to nutritious food, were not sexually potent. Many men reported that nutritious food could produce more semen to make men as *birjoban purush*, where *birjo* means semen, *birjoban* means someone rich in *birjo*, and purush means man. *Birjoban*, thus, also indicates sexually powerful. Therefore, *birjoban purush* means a sexually ‘powerful’ or ‘potent’ man. Men who had less income reported their frustration about physical and sexual weaknesses which might have resulted from their less intake of nutritious food. Some men compared the whitish colour of semen with the white part of the egg and reported eating eggs especially ‘bigger’ duck eggs regularly to produce more semen. Fat rich in fat, such as *ghee*, was considered good for the production of semen.

Many believed that poor men have poor quality of semen too. The poor quality of semen could result in the birth of children with poor physical and mental growth. Essentially, their belief was that the quality of semen determines the intellectual quality of children. A key-informant stated: “semen contains seed. For a good-quality tree, you need good quality of seeds; similarly, if you want to have good children, you need good quality of semen.” Like this key-informant, many men had a belief that “a Judge cannot be born in a poor family as one cannot overcome the genetic influence which passed through semen.” If the quality of semen is ‘bad’, children may be born with mental dullness or can have other physical defects. A child in a poor family infrequently achieves success in professional or family life.

Some young men were concerned about the small quantity of their semen per ejaculation. An ‘adequate’ amount of semen was described as symbolizing *birjoban purush*. One unmarried man stated that he and his friend had measured the amount of ejaculated semen after masturbation and evaluated their sexual potentia-lity. Most men were unaware of any standard amount of discharged semen per ejaculation. An unmarried man stated: I ejaculate a small amount of semen. I have seen people in pornographic movies ejaculate a huge amount of semen. Probably, I have a shortage of semen. I have to marry quickly. Otherwise, after marriage, I will have a shortage of semen.

These men were terrified of lacking a sufficient amount of semen. They believed only a finite amount of semen could be stored in the male body. Men reported that, due to semen loss in ‘illegal’ (e.g. pre-marital or extra-marital sex) or ‘unnatural’ (masturbation) ways, storage of semen could be depleted and become diluted. Thus, semen could be automatically discharged while urinating or defaecating which resulted from sexual weakness and diseases making men less masculine.

Some newly-married men were concerned about the small amounts of their ejaculated semen. They reported that their semen gradually decreased in amount after marriage due to its regular discharge. They were frightened and began taking more nutritious food to increase the production of semen to ensure fatherhood.

### Seminal discourse: perspectives of medical practitioners

Traditional practitioners described ‘whitish discharge’ of men as *meho* and *pro-meho*, a condition analogous to gonorrhoea. Some men reported being familiar with these terms which were mentioned in the leaflets of traditional practitioners. A few young men reported experiencing this problem during urination.

I sometimes pass semen-like substances during urination in the morning which looks like a thin thread. When I get up in the morning and go for urination, semen-like white fluid passes either before or after urination (*chikon dharai ber hoi*). I must be sexually weak. If this continues, stock of my semen will be finished. What will I do in my married life? One doctor has told me to drink less water. Now I am even afraid to urinate. Another doctor said, I had to drink more water. What should I do? Would you kindly suggest me where should I go for treatment?

This young man perceived serious physical problems needing appropriate medical investigation. Although he visited doctors, the advice he gained was inconsistent, confusing, and contradictory. His perceptions of decreasing body storage of semen due to passing urine and becoming sexually weak cannot only be analyzed as the sexual health problem of an individual originating from lack of knowledge. We need to understand that men's concerns regarding semen storage and its depletion are culturally implanted. Traditionally, semen is considered a source of physical and sexual potency for men, further reinforced by the advertisements of traditional practitioners.

Traditional practitioners described nocturnal emissions as symptoms of sexual diseases and *dhatu durbolota*. Their advertising leaflets claim: “*Swapno-dosh* is an outcome of excessive *hasto-moithun* (masturbation) in young life.” Although men knew that it might happen, they worried about its ‘normality’, and the ‘normal’ frequency remained a big question to many men.

I went to a village doctor to get rid of *swapno-dosh*. He stated that it happens due to too much sexual thinking, and it is a sexual disease. He asked me not to think about sex. Believe me, despite having any sexual thoughts, it often happens. I explained, but the doctor did not believe. He laughed and asked me to marry soon. If I lose semen in this way, I will have no semen left for my wife. It makes me worried. I heard that my friends had *swapno-dosh* hardly once a month or less than that. Why do I have so frequent *swapno-dosh*?

We discussed the issue of semen loss with both allopathic STD specialists and traditional practitioners in both urban and rural areas. Allopathic physicians dismissed common claim of men that masturbation leads to problems of semen loss. Traditional practitioners supported this belief. One traditional practitioner claimed, “infrequent masturbation had no significant negative impact on health of men, but if performed ‘too much’, it definitely affects health through semen loss.” The terms ‘infrequent’ and ‘too much’ had different meanings to different practitioners. ‘Infrequent’ meant engaging in masturbation once a week or twice a month. The meaning of ‘too much’ was also diversely reported, ranging from three to five times a week.

There is a limit of production of semen in human body. If a young man is involved in masturbation, his semen, the vital source of energy, will be lost before marriage. Therefore, he will suffer from general weakness, and there are many other side-effects. He may have lost his memory, may not concentrate in his studies, and have acne on his face, and his eyes may be shrunken. To produce semen, a person requires taking rich food, and in our poor society, it is not possible for many men to eat nutritious food, so production of semen is hampered. Many may be infertile in future life. Moreover, masturbation is religiously considered a sinful act. Men who engage in it suffer from guilt which is bad for his overall health and well-being. He cannot be able to be a productive citizen.

Traditional practitioners stated that young men who indulged in ‘excessive’ masturbation lose semen, resulting in loss of memory, weakness, indigestion, and palpitations. These beliefs and propositions were described in the leaflets of traditional practitioners for wide dissemination. Many men self-diagnosed their physical problems as a result of semen loss. A traditional practitioner claimed:

Allopathic doctors only know about syphilis and gonorrhoea, they have few specific antibiotics to treat every problem, and they do not know much about sexual concerns of men and pay little attention to semen loss. Our knowledge is based on reported concerns of men.

Many men visited these practitioners more often than allopathic doctors on the following grounds:

I first visited an *ayurvedic* doctor and took his medicines for few months without any significant improvement. Then I went to a ‘modern’ sex specialist in the city and became more depressed. I found that he did not understand my problem. He thought that I was suffering from mental problem. He stated that everything was fine with me. He gave me some vitamin tablets, …is not that strange? He labelled me as a psychiatric patient. Then I returned to my *ayurvedic* doctor, at least he understood my problem.

These statements reflect how men internalized their problems in the context of two different discourses of medical practitioners. The advertisements of traditional practitioners were distributed in the form of leaflets in various community settings, transport stations, market places, and parks. These advertisements describe how men who spend ‘energy’ through expending semen by masturbation in their youth (*joubone autiricto sakti khai*) would subsequently suffer from various sexual health problems. These advertisements claimed to treat various male sexual health problems with a ‘money-back guarantee’. All these advertisements identified masturbation as the main cause of semen loss, resulting in depletion of semen, thinning of semen, deformed size and shape of penis, lacking sexual power, and failure of erection and prolonged intercourse.

## DISCUSSION

Men in this study also referred to semen as *dhatu*. The word *dhatu* also means ‘vital essence’ which is analogous to *birjo*. The Bangla word *birjo* used to mean semen. They also used the word *mal* (valuable goods) to indicate semen. Thus, the symbolic meaning of *birjo* is wealth and power of men. Non-STI sexual health concerns of men, particularly semen loss, cannot be properly analyzed and understood through biomedical perspectives that ignore the sociocultural construction of masculine sexuality where semen is the symbol of prestige and power of men. Concerns of men are deeply embedded in the ethnomedical understanding of semen in cultural and historical perspectives, reflecting tensions of masculine sexuality in the patriarchal society.

We think that it is appropriate to describe the *dhat* syndrome of women to further contextualize men's perceived whitish discharge in the understanding of semen loss since we argue that both men and women share some similarities in experiencing their concerns about their sexual health. Indian researchers found that a significant proportion of women suffer from the *dhat* syndrome (36, 46, 47). In Bangladesh, women perceive passage of non-pathological whitish discharge per vagina as a pathological event (23, 48). Like men, women also perceive such discharge as loss of *dhatu*, a vital fluid like semen, necessary for health and the well-being of women. Researchers argue that perceived symptoms of women were generally somatic in nature (23). Allopathic practitioners often miss the broader meanings of both vaginal discharge and semen loss.

We agree with other researchers that the way traditional *ayurvedic* practitioners interpret the cultural messages of vaginal ‘whitish’ discharge reflects concerns of women more closely (36, 47, 49, 50). For example, Nichter argues the expression of tension regarding vaginal secretion is a consequence of the powerless situation of women in many aspects of life (49). One may argue that Nichter's proposition of women's powerlessness cannot be applied to men as they are socioculturally placed in privileged and dominating positions in Bangladeshi society. However, we argue that men are also ‘powerless’ in some aspects of life. For example, the power of men in terms of sexual skills and performance is an ongoing challenge. Men are concerned about their perceived ‘powerless’ situation in their sexual life which is expressed through various tensions of sexual health, including semen loss. This is a sex-related culture-bound syndrome which may have similarities with other psychosexual and behavioural cases. Koro in India is a condition where men complain that their penis has shrunk because of tensions relating to their penis, sex, and related constraints of life (51). Thus, concerns of men about semen loss like that of ‘whitish discharge’ of women could be a way of communicating psychosocial concerns of men, a somatic idiom for depression, expressed through bodily secretion.

Sexual health concerns of men may not always reflect biomedical realities as the meanings are deeply embedded in the sociocultural context of male-dominated patriarchal societies where sexual power and potency of men are considered to be a valuable asset for men, families, societies, and the state. Philaretou and Allen claim: “male sexual anxiety can result from dysfunctional meanings associated with socialization into a mechanistic masculine script of toughness, competitiveness, autonomy, and hypersexuality” (3). Men's sense of alienation affects their sexual life in the industrial and post-industrial societies where men often fail to prove their occupational and economic achievements (3, 52, 53).

The economy of Bangladesh is still mainly based on agriculture with industrialization in an initial state. Widespread poverty and massive unemployment remain the salient features in the life of most people. Bangladeshi men traditionally equate monetary wealth with manhood and sexual potency (54). To emphasize the significance of semen in the life of men, traditional practitioners in India use the following metaphor that “a poor man who has no money” is similar to “a sexually weak person who has no semen” (4). In the context of economic crisis in Bangladesh, the considerable struggle of men for economic survival is extended to their sexual life, encouraging men to feel the need to preserve sufficient amounts of semen. Involvement of Bangladeshi men in encountering overall challenges for better survival complicates their day-to-day life, including sexual activities and desires. These challenges affect men further reflected through their concerns about semen loss, symbolic threats for masculine men, to show manly capacity in every aspect of life. We support the research findings of Peter Aggleton who states that, “to lose too much semen in the wrong kind of way may be to have one's sense of masculinity and manhood threatened” (55). Analogous to monetary power and manhood, men also considered being *birjoban* in acquiring good quality of semen which was vital for reproductive success of men and was perceived necessary to carry on the patriarchal heredity for producing healthy and meritorious offspring for family, society, and the state.

Perceptions of medical practitioners about the vitality of semen cannot be seen in medico-historical cultural isolation. In *ayurvedic* and Chinese medical histories, semen has been portrayed as an ‘essence’ of men's life (2). The South Asian concepts of semen are grounded in similar frameworks of the ancient *ayurvedic* and Chinese understanding of semen (19, 56). Sixty to 100 drops of blood are required to produce one drop of semen (4, 57). Some *ayurvedic* texts state that each sexual intercourse is equivalent to an energy expenditure of 24 hours of mental work or 72 hours of physical work (58). It is believed that consumption of 60 pounds of food is required to produce the amount of semen in a single ejaculation. Scholars have argued that the cultural meanings of semen loss, especially in South Asian countries, can be compared with the 18th century Western medical propositions. The depletion of semen, the vital fluid of body and blood, was thought to lead to insanity. “The loss of one ounce of it [semen] enfeebles more than forty ounces of blood (59). Semen is important for healthy bodily functions, and wasting of semen in ‘unnecessary’ sexual activities, especially for self-pleasure without the need of reproduction, can cause illness. Tissot argues that sperm is the ‘end-pro-duct of digestions’, ‘essential ointment’, and the ‘leading liqueur’ (59). In Victorian times, between the 1800s and early 1900s, physicians also believed in the harmful impacts of semen loss on health (60). Therefore, these views are historically grounded, and physicians of the current era also believe in this.

### Eradication of genital infections versus improving sexual health: a dilemma

“The fact that much modern medicine continues to understand these problems [non-STIs sexual health problems] largely within the rubric of myths and misconceptions” which encourage allopathic practitioners to disregard concerns of men about sexual health (5). The findings of this study demonstrate that men's understanding of their sexual health concerns and perspective of allopathic practitioners in treating STIs is not linearly positioned. Bhatia and Malik reported that around 43% of the Indian male population who dropped out from the sexual health clinic were not satisfied with the explanation that semen loss was not harmful, and their symptoms were psychological (7). Supporting findings of Singh (30), our participants reported incongruity with doctors’ understandings of their problems. It is inappropriate to blame men and challenge their culturally-implanted beliefs in the domain of positivist knowledge of biomedical science.

Current STI/HIV programmes do not address men's perceived concerns about sexual health. Men do not share the same concerns about STIs/HIV as do public-health programmes. This causes a split between public health and men's own priorities. Most STI clinics in Bangladesh receive a significant proportion of males with psychosexual problems, although they do not offer expert professional services to these clients (1, 61). These men sought medical help from unregulated and unskilled private practitioners who were considered to have a better understanding of male sexual health problems (1).

The donors generally prefer to find a cost-effective way to eradicate genital infections ignoring non-transmissible sexual health problems. Thus, the current programmes are donor-driven and predominantly designed to address the problems of STIs, including HIV (1). It seems that we focus on genital infections, not on the owners of the genitals. The findings of many other studies reveal that reported sexual unhappiness of men, confusion, and suffering of low self-esteem influence sexual acts and relationships of men with their sex partners, resulting in unhealthy and unhappy sexual life and poor compliance to family-planning activities (4, 54).

Genital discharge suggests the presence of sexual and/or reproductive tract infections. Currently, in South Asian countries, including Bangladesh, STIs are treated by the syndromic management approach based on the recognition of particular sets or combinations of symptoms and signs by primary healthcare workers (62, 63). Confusion exists among healthcare workers who are not adequately trained to distinguish between perceived genital discharge of patients (semen loss during or after urination) and pathological discharge due to STIs (or RTIs) (1, 2, 16).

The prevalence of clinically-significant RTIs among women in South Asian countries, including Bangladesh, is lower than was previously thought (23). Similarly, Hawkes reported that reported symptoms of burning urination and swelling of testes of Bangladeshi men do not necessarily indicate the presence of STIs (14). In other parts of Asia, such as in India and Pakistan, men's complaints of burning sensation during urination and penile discharge have led medical practitioners using the syndromic approach to misdiagnose and over-treat as gonorrhoea and chlamydia (1, 5, 21). Therefore, the chance of over-estimation of STIs through self-reports and resultant over-treatment cannot be ignored (23).

However, men who report semen loss through urine may have STIs. Therefore, it would not be appropriate to interpret the complaint of semen loss from only cultural perspectives and avoid treatment. Medicalizing psychosocial and psychosexual concerns of men about semen loss increases the chance of ignoring the cultural, psychosocial and metaphorical meanings (36). This may increase the chance of over and/or inappropriate treatment on the one hand and discourage men to seek treatment from the appropriate health facilities on the other.

## CONCLUSION

The meanings of semen and sexual intercourse and related sexual health problems are deeply rooted tensions in the life of men. Concerns of men when viewed as the individual's subjective disbeliefs, myths, or misconceptions may lead to missing opportunities to properly understand these problems for appropriate interventions. Historically, semen is seen as the most powerful body fluid and an asset of men. Sperm in semen is necessary to produce human offspring, keeping the heredity and blooming life on this earth. Now semen can be infected with HIV causing AIDS, the deadly socioclinical condition destroying human civilization and development across nations.

Men are blamed for their unfaithful sexual behaviour as “without men there would be no epidemic [HIV]” (64). Men are told not to ejaculate inside their partners’ body to prevent disease reminding them that semen contains virus, not vitality. Nevertheless, men are not too concerned about a virus. They are rather persistently concerned about the essence of semen, its vigour, quality, and quantity for being accredited as a ‘sexually potent’ man. Seminal discourse of men, which accepts vitality and rejects virus, terrifies them for any unproductive semen loss. In this paper, we argue that public-health perspectives of eradicating STIs/HIV do not correspond with lay concerns of men about their sexual health and may actually lessen male involvement in sexual and reproductive health interventions. We propose a paradigm shift of HIV interventions towards improving the quality of sexual health of men (and women). In such a shift, the discourse of semen centres on vitality and not on virus. Sexual ill-health imposes great burdens on health services and creates an unhealthy society in terms of sexual well-being, the severity of which cannot be measured by conventional scales relating to positivist science.

Addressing perceived concerns of men about sexual health can be considered ‘an effective entry point’ to both reproductive and sexual health programmes (5). However, “if men can be convinced that health care professionals understand their problems, they may be drawn into more active roles in relation to women's reproductive health as well” (4). Therefore, the challenge yet persists in increasing the understanding of sexual health concerns of both men and women and to integrate the sociocultural meanings of these concerns into the current sexual and reproductive health-delivery system of Bangladesh and other developing countries.

## ACKNOWLEDGEMENTS

This paper is based on data of a project conducted at ICDDR,B with support from Edith Cowan University, Perth, Western Australia and AusAID research grants. The authors extend their thanks to the Social and Behavioural Sciences Unit of the Public Health Sciences Division for support to the project. M. Shamsul Islam Khan, the Managing Editor of the Journal of Health, Population and Nutrition and Head, Publications Unit of ICDDR,B must be gratefully thanked for his editorial support. All project staff members are to be thanked for their hard work. Finally, the authors express their gratitude to the study participants for their generous support and participation throughout the project period.
